# Early high-dose intravenous methylprednisolone is associated with faster symptom improvement in persistent ocular myasthenia gravis: a single-center retrospective study

**DOI:** 10.3389/fneur.2026.1956054

**Published:** 2026-10-01

**Authors:** Noritaka Matsuo, Genya Watanabe, Chihiro Sasaki, Ayane Tamagake, Moeko Saito, Kenichi Tsukita, Yasushi Suzuki

**Affiliations:** Department of Neurology, National Hospital Organization Sendai Medical Center, Sendai, Japan

**Keywords:** intravenous high-dose methylprednisolone, MG-ADL, minimal symptom expression, ocular myasthenia gravis, oral corticosteroid

## Abstract

**Background:**

High-dose intravenous methylprednisolone pulse therapy (HMP) may provide rapid symptom relief in ocular myasthenia gravis (MG), but the clinical impact of its early use as induction immunotherapy remains unclear. We evaluated whether early HMP was associated with more rapid symptom improvement in patients with persistent ocular MG.

**Methods:**

This single-center retrospective study included 36 patients with ocular MG who received corticosteroid-based immunotherapy and remained classified as having ocular MG throughout 2 years of follow-up. Patients were categorized according to their initial immunotherapy strategy into an early HMP induction group (*n* = 19), which received HMP at oral prednisolone initiation or within 3 weeks thereafter, and an oral corticosteroid (OCS) induction group (*n* = 17), which initially received OCS without HMP. Additional HMP during follow-up was permitted. The primary outcome was the longitudinal change in ocular MG Activities of Daily Living (MG-ADL) score over 24 months, analyzed using a linear mixed-effects model.

**Results:**

Baseline ocular MG-ADL scores were higher in the HMP group (mean ± standard deviation, 4.842 ± 1.642 vs. 3.353 ± 1.057). The mixed-effects model demonstrated a significant group-by-time interaction (*p* < 0.001). At 1 month, the least-squares mean ocular MG-ADL score was 0.474 (95% confidence interval [CI], 0.024–0.923) in the HMP group and 2.000 (95% CI, 1.525–2.475) in the OCS group, with a significant between-group difference after Tukey adjustment (*p* < 0.001). From 3 months onward, scores remained low in both groups without significant between-group differences. The HMP group achieved minimal symptom expression earlier. Oral prednisolone doses and prednisolone-related adverse events did not differ significantly between groups. Initial worsening occurred in two HMP-treated patients, but neither developed myasthenic crisis.

**Conclusion:**

In persistent ocular MG, early HMP as part of induction immunotherapy was associated with faster ocular symptom improvement and earlier achievement of minimal symptom expression than OCS induction alone, while long-term clinical outcomes and oral prednisolone doses were similar. Early HMP may be useful when rapid symptom control is required, although prospective studies are needed.

## 1 Introduction

Myasthenia gravis (MG) is an autoimmune disorder of the neuromuscular junction characterized by autoantibodies against postsynaptic targets, including the acetylcholine receptor (AChR) and muscle-specific kinase (MuSK). The prevalence of MG has increased over recent decades, largely due to advances in diagnostic methods and population aging, and is currently estimated at approximately 10–20 per 100,000 population in many countries. A similar trend has been reported in Japan, where the proportion of older patients with elderly-onset MG has increased (1). Ocular symptoms represent the initial manifestation in approximately 50–60% of patients with MG, whereas approximately 15–20% remain classified as having ocular MG without developing generalized disease during follow-up (2, 3). Ocular MG is characterized by weakness confined to the extraocular muscles and the levator palpebrae superioris, with ptosis and diplopia as the predominant clinical manifestations. Although ocular MG is generally considered less severe compared with generalized MG, persistent ocular symptoms can substantially impair daily functioning, visual performance, work productivity, and quality of life.

In ocular MG, cholinesterase inhibitors are recommended as the initial symptomatic treatment, whereas corticosteroid-based immunotherapy is introduced when symptom control remains inadequate (1, 4). Oral prednisolone (PSL) has been reported to be effective in ocular MG (2, 5), and the European Federation of Neurological Societies (EFNS) and European Neurological Society (ENS) guidelines recommend initiating treatment at 10–20 mg/day, followed by dose escalation until symptom control is achieved (4). However, prolonged oral corticosteroid (OCS) therapy may be limited by adverse effects, including glucose intolerance, osteoporosis, infection, psychiatric symptoms, and cosmetic changes. These concerns have become increasingly important in Japan owing to the growing proportion of older patients with MG. Consequently, current treatment strategies emphasize the rapid achievement of favorable clinical outcomes while minimizing cumulative corticosteroid exposure.

The 2022 Japanese clinical guidelines for MG identify achievement of minimal manifestations (MM) or better with an oral PSL dose of ≤5 mg/day (MM-5 mg) as an important practical treatment target (1). This recommendation reflects the principle that sustained disease control should be achieved while minimizing long-term corticosteroid exposure. Minimal symptom expression (MSE), defined as a total MG-Activity of Daily Living (ADL) score of 0 or 1, has also been proposed as a patient-reported treatment target for MG (6). Collectively, these outcome measures emphasize both physician-assessed disease status and the patient’s perception of symptom burden in daily life.

High-dose intravenous methylprednisolone pulse therapy (HMP) has long been used as a rapid-acting treatment for generalized MG (7), and its efficacy in ocular MG has also been suggested (8–10). Rapid clinical improvement may be achieved with HMP while subsequent treatment is maintained with relatively low doses of oral PSL, making this approach well suited to the current treatment paradigm that prioritizes early achievement of MM-5 mg and MSE. However, whether early induction of HMP facilitates more rapid symptom control and reduces subsequent oral PSL exposure in patients with ocular MG remains unclear. Therefore, the present study aimed to evaluate the efficacy of HMP as induction immunotherapy for ocular MG and assess its potential steroid-sparing effects.

## 2 Materials and methods

### 2.1 Patient selection and treatment groups

This single-center retrospective observational study included all patients newly diagnosed with MG in our department between April 2019 and June 2024. MG was diagnosed according to Japanese clinical guidelines (1). Clinical diagnosis was based on fluctuating and fatigable symptoms, including ptosis, ophthalmoplegia, and facial weakness, together with serological or neurophysiological evidence of neuromuscular junction dysfunction. Anti-AChR antibodies were measured by radioimmunoassay, and a value of > 0.2 nmol/L was considered positive. Repetitive nerve stimulation was performed to assess the neuromuscular junction dysfunction by delivering 10 consecutive stimuli at a frequency of 3 Hz; a decremental response of ≥ 10% was considered positive. Single-fiber electromyography was not performed.

During the study period, 188 patients were newly diagnosed with MG in our department, including 71 diagnosed with ocular MG at the initial visit. Patients with < 2 years of follow-up at our hospital, prior immunotherapy at another institution, no immunotherapy, an initial treatment strategy other than the corticosteroid-based strategies evaluated in this study, or secondary generalization within the 2-year follow-up period were excluded. Of the 12 patients categorized as receiving “other treatments” in Figure 1, 10 were managed with pyridostigmine alone and did not receive immunotherapy during follow-up, whereas 2 received tacrolimus in combination with pyridostigmine without corticosteroid-based induction. Six patients who developed generalized MG during the 2-year follow-up period were excluded from subsequent analyses. Consequently, 36 patients who remained classified as having ocular MG throughout the 2-year follow-up period were included in the analysis.

**Figure 1:**
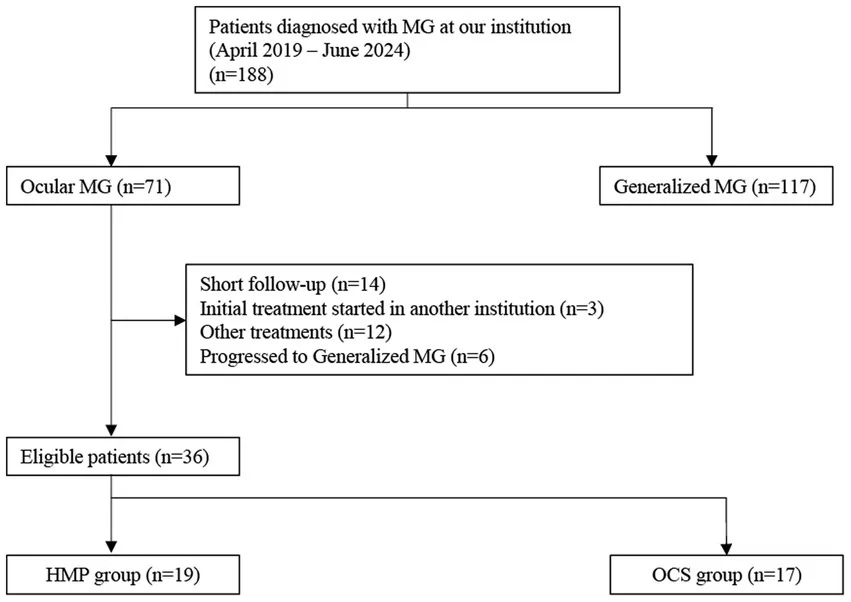
Flowchart of patient selection. A short follow-up period was defined as less than 24 months. Other treatments comprised pyridostigmine alone without immunotherapy (*n* = 10) and tacrolimus plus pyridostigmine without corticosteroid-based immunotherapy (*n* = 2). MG, myasthenia gravis; HMP, high-dose methylprednisolone pulse therapy; OCS, oral corticosteroid therapy.

All included patients received oral PSL as corticosteroid-based immunotherapy. The patients were categorized into two treatment groups according to the initial immunotherapy strategy. The HMP group comprised 19 patients who received HMP at the initiation of oral PSL or within 3 weeks thereafter as induction therapy. The OCS group comprised 17 patients who initially received OCS therapy without concomitant HMP during the induction period, defined as the first 3 weeks after oral PSL initiation. Additional HMP could be administered during follow-up when clinically indicated, and patients were analyzed according to their initial treatment strategy. The treatment-related exclusion criteria were applied to the initial treatment strategy; concomitant or subsequent use of calcineurin inhibitors (CNIs) after initiation of OCS or HMP did not constitute an exclusion criterion. The first HMP course consisted of intravenous methylprednisolone at 1,000 mg/day for 3 consecutive days. Additional HMP courses were administered according to clinical response, with more than three courses administered when clinically indicated, and subsequent courses could be abbreviated or dose-reduced at the treating physician’s discretion (Supplementary Table S4). This study was approved by the Institutional Ethics Committee of the National Hospital Organization Sendai Medical Center (approval no. rin26-8 [jin]). The requirement for written informed consent was waived owing to the retrospective observational study design. Instead, an opt-out option was provided on the institutional website.

### 2.2 Outcome measures

The baseline variables included age at onset, sex, antibody status, duration from symptom onset to initiation of immunotherapy, treatment details, and disease severity before and after treatment. All patients were followed up for at least 2 years after treatment initiation. Disease severity was assessed using the Myasthenia Gravis Activities of Daily Living scale (MG-ADL) (11). Ocular symptom severity was evaluated using the ocular MG-ADL score, which comprised the diplopia and ptosis items of the MG-ADL. Each item was scored from 0 to 3, yielding a total ocular MG-ADL score ranging from 0 to 6. MSE was defined as an MG-ADL total score of 0 or 1 (6).

The primary outcome was the longitudinal change in the ocular MG-ADL score up to 24 months following treatment initiation. The ocular MG-ADL score was assessed at baseline and 1, 3, 6, 12, and 24 months. Secondary outcomes included the time from treatment initiation to the first achievement of MSE, postintervention status according to the Myasthenia Gravis Foundation of America postintervention Status (MGFA-PIS) (12), longitudinal changes in oral PSL dose, initial worsening following HMP (13), and adverse events related to oral PSL.

### 2.3 Statistical analysis

Statistical analyses were performed using Python (version 3.12.13) on Google Colaboratory. Continuous variables were expressed as the mean ± standard deviation or median (interquartile range), as appropriate. Continuous variables were compared using Welch’s t-test or the Mann–Whitney U test, as appropriate, whereas categorical variables were compared using Fisher’s exact test. Longitudinal changes in ocular MG-ADL scores were analyzed using a linear mixed-effects model with treatment group, time, and the group-by-time interaction as fixed effects and a patient-specific random intercept. Time was treated as a categorical variable, with baseline as the reference. The overall group-by-time interaction was evaluated using a likelihood-ratio test, and least-squares means with 95% confidence intervals were estimated for each group at each time point. Pairwise comparisons of least-squares means were adjusted for multiple comparisons using Tukey’s method.

Time to MSE was analyzed using the Kaplan–Meier method, and between-group differences were assessed with the log-rank test. All statistical tests were two-sided, and a *p*-value of <0.05 was considered significant.

## 3 Results

The baseline characteristics of the two groups are summarized in Table 1. The ocular MG-ADL score at initiation of immunotherapy was significantly higher in the HMP group compared with the OCS group (4.8 ± 1.6 vs. 3.4 ± 1.1, *p* = 0.003). The diplopia subscore was also higher in the HMP group (2.3 ± 1.1 vs. 1.3 ± 1.0, *p* = 0.010), indicating more severe diplopia at baseline. By contrast, sex, age at onset, anti-AChR antibody positivity, and the presence of thymoma did not differ significantly between the groups. Among the 19 patients in the HMP group, 9 received one HMP course, 3 received two courses, and later courses were abbreviated or dose-reduced in two patients (Supplementary Table S4). CNIs were used in 9 of 19 patients (47.4%) in the HMP group and 9 of 17 (52.9%) in the OCS group (*p* = 1.000). The median time from immunotherapy initiation to CNI initiation was 0 days (interquartile range [IQR], 0–7) in the HMP group and 1 day (IQR, 0–52) in the OCS group (*p* = 0.677) (Table 1).

**Table 1:** Clinical characteristics of the HMP and OCS group.

Characteristic	HMP group (*n* = 19)	OCS group (*n* = 17)	*p*-value
Male, *n* (%)	14 (73.7)	11 (64.7)	0.721
Age at onset, year	60.4 ± 12.0	60.1 ± 20.9	0.958
Time from disease onset to immunotherapy, days	94.0 [75.0, 260.5]	149.0 [57.0, 504.0]	1.000
Baseline ocular MG-ADL score	4.8 ± 1.6	3.4 ± 1.1	0.003
Baseline diplopia score	2.3 ± 1.1	1.3 ± 1.0	0.010
Baseline ptosis score	2.6 ± 0.8	2.1 ± 0.9	0.100
Thymoma, *n* (%)	1 (5.3)	1 (5.9)	1.000
Anti-AChR antibodies, *n* (%)	19 (100.0)	17 (100.0)	1.000
CNI use, *n* (%)	9 (47.4)	9 (52.9)	1.000
Time to CNI initiation, days	0 [0–7]	1 [0–52]	0.677

Values are expressed as *n* (%), mean ± standard deviation, or median [interquartile range]. Age and baseline ocular MG-ADL, diplopia, and ptosis scores are presented as mean ± standard deviation; time from disease onset to immunotherapy and time to CNI initiation are presented as median [interquartile range]. *p*-values were calculated using Welch’s *t*-test, the Mann–Whitney *U*-test, or Fisher’s exact test, as appropriate. HMP, high-dose methylprednisolone pulse therapy; OCS, oral corticosteroid therapy; MG-ADL, Myasthenia Gravis Activities of Daily Living profile; AChR, acetylcholine receptor; CNI, calcineurin inhibitor.

Longitudinal analysis of the primary outcome using a linear mixed-effects model demonstrated a significant group-by-time interaction (likelihood-ratio χ^2^ = 45.75, df = 5; *p* < 0.001), indicating that the trajectory of ocular MG-ADL scores differed between the treatment groups. At baseline, the least-squares mean ocular MG-ADL score was 4.842 (95% CI, 4.393–5.291) in the HMP group and 3.353 (95% CI, 2.878–3.828) in the OCS group (Tukey-adjusted *p* < 0.001). At 1 month, the estimated score decreased to 0.474 (95% CI, 0.024–0.923) in the HMP group, compared with 2.000 (95% CI, 1.525–2.475) in the OCS group (Tukey-adjusted *p* < 0.001). At 3, 6, 12, and 24 months, the estimated scores were 0.368 (95% CI, −0.081 to 0.818) versus 0.588 (95% CI, 0.113–1.063), 0.263 (95% CI, −0.186 to 0.713) versus 0.294 (95% CI, −0.181 to 0.769), 0.368 (95% CI, −0.081 to 0.818) versus 0.412 (95% CI, −0.063 to 0.887), and 0.368 (95% CI, −0.081 to 0.818) versus 0.353 (95% CI, −0.122 to 0.828), respectively, in the HMP and OCS groups; no significant between-group differences were observed at these time points (all Tukey-adjusted *p* > 0.999) (Figure 2). Observed mean ± standard deviation values at each time point are shown in Table 2.

**Figure 2:**
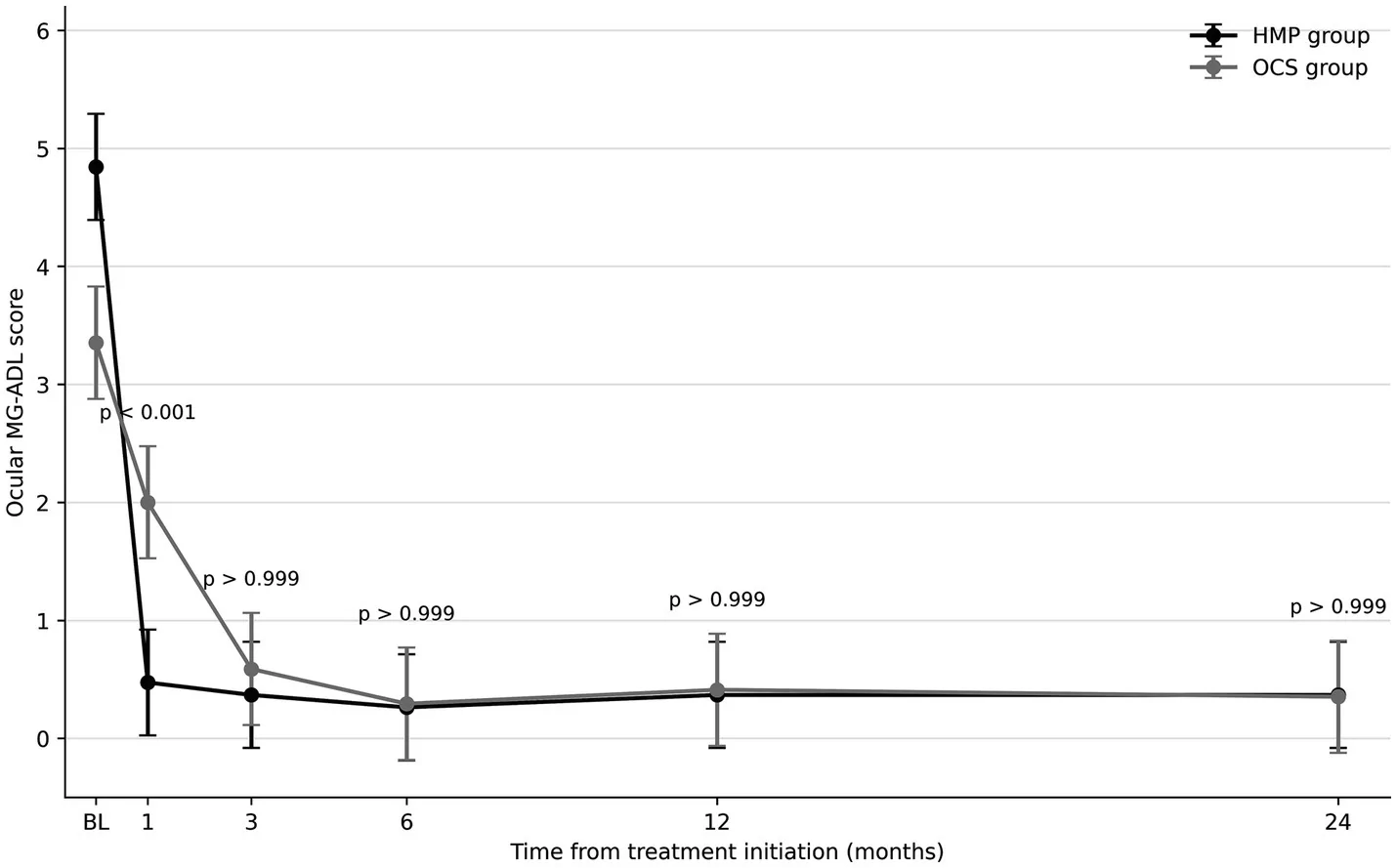
Longitudinal changes in ocular MG-ADL scores estimated using a linear mixed-effects model. Points represent least-squares means, and error bars indicate 95% confidence intervals. *p*-values indicate Tukey-adjusted pairwise comparisons between the HMP and OCS groups at each post-baseline time point. The black line indicates the HMP group, whereas the gray line indicates the OCS group. BL, baseline; HMP, high-dose methylprednisolone pulse therapy; OCS, oral corticosteroid therapy; MG-ADL, Myasthenia Gravis Activities of Daily Living.

**Table 2:** Observed ocular MG-ADL scores during follow-up.

Group	Baseline	1 month	3 months	6 months	12 months	24 months
HMP group (*n* = 19)	4.842 ± 1.642	0.474 ± 0.905	0.368 ± 0.684	0.263 ± 0.562	0.368 ± 0.831	0.368 ± 1.012
OCS group (*n* = 17)	3.353 ± 1.057	2.000 ± 1.414	0.588 ± 1.121	0.294 ± 0.588	0.412 ± 0.870	0.353 ± 0.702

Values are expressed as observed mean ± standard deviation. Longitudinal changes were analyzed using a linear mixed-effects model as described in the Statistical Analysis section. HMP, high-dose methylprednisolone pulse therapy; OCS, oral corticosteroid therapy; MG-ADL, Myasthenia Gravis Activities of Daily Living.

The Kaplan–Meier curves for the time to MSE are shown in Figure 3. The log-rank test demonstrated a significant between-group difference, with patients in the HMP group achieving MSE earlier than those in the OCS group. However, the cumulative MSE achievement rate over the 2-year follow-up period did not differ significantly between the groups.

**Figure 3:**
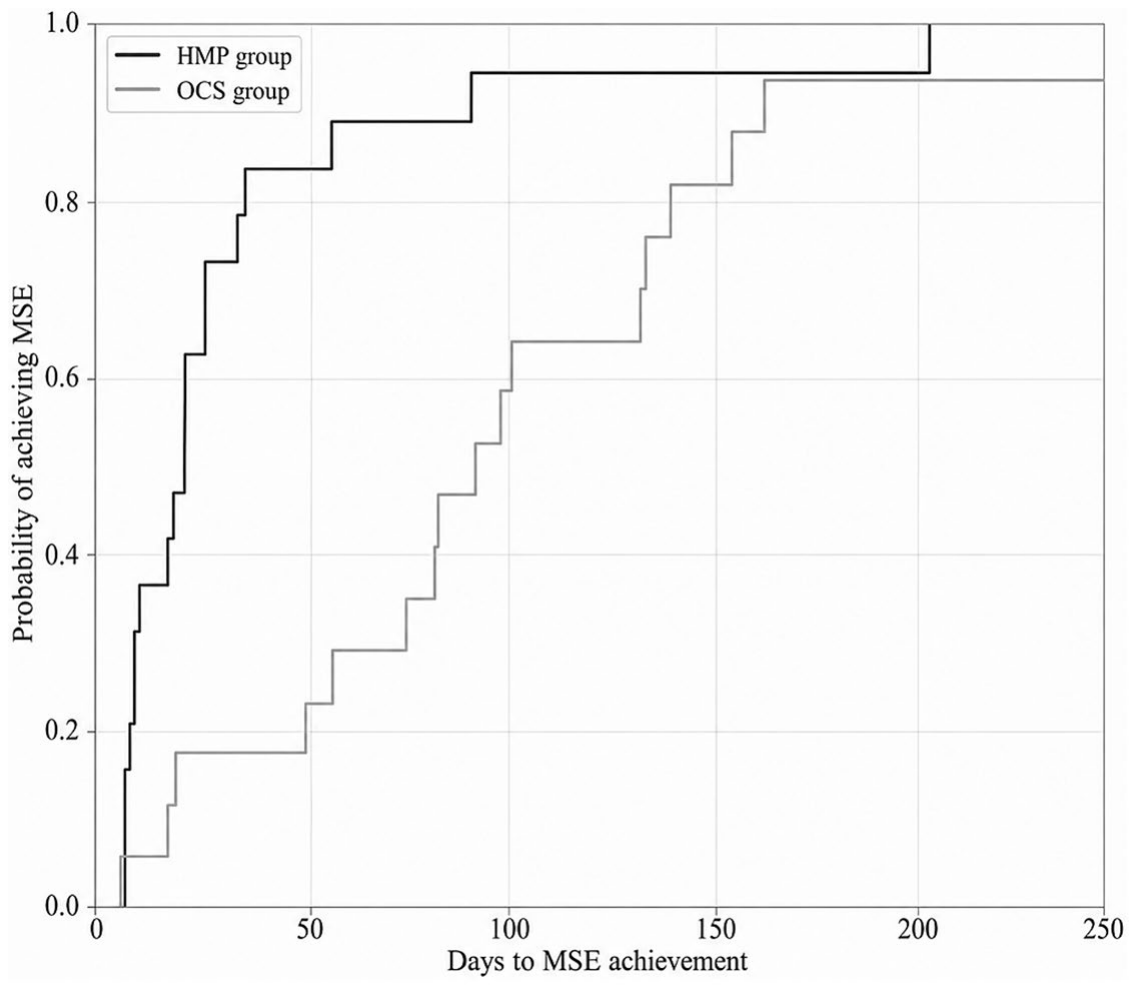
Time to achievement of minimal symptom expression following immunotherapy. Kaplan–Meier curves show the cumulative probability of achieving minimal symptom expression in the HMP and OCS groups. The black line indicates the HMP group, whereas the gray line indicates the OCS group. Between-group differences were assessed using the log-rank test. HMP, high-dose methylprednisolone pulse therapy; OCS, oral corticosteroid therapy.

During follow-up, additional HMP was administered to 4 of the 17 patients initially assigned to the OCS group. Two patients achieved MSE before receiving additional HMP, whereas the remaining two achieved MSE after HMP administration. Sensitivity analyses excluding these four patients as well as analyses censoring the two patients who achieved MSE after additional HMP at the time of HMP initiation yielded consistent results. Earlier achievement of MSE in the HMP group was maintained in both analyses (Supplementary Table S1). Among the six patients who developed secondary generalization, two had received HMP as the initial immunotherapy strategy, two had received OCS, and two had been managed with pyridostigmine alone without immunotherapy before generalization (Supplementary Table S3).

A similar pattern was observed for the MGFA-PIS (Figure 4). At 1 month, the proportion of patients who achieved MM or better was significantly higher in the HMP group, indicating more rapid clinical improvement. However, no significant between-group differences in the proportion of patients achieving MM or better were observed from 3 months onward, and both groups ultimately achieved favorable clinical status during the 2-year follow-up.

**Figure 4:**
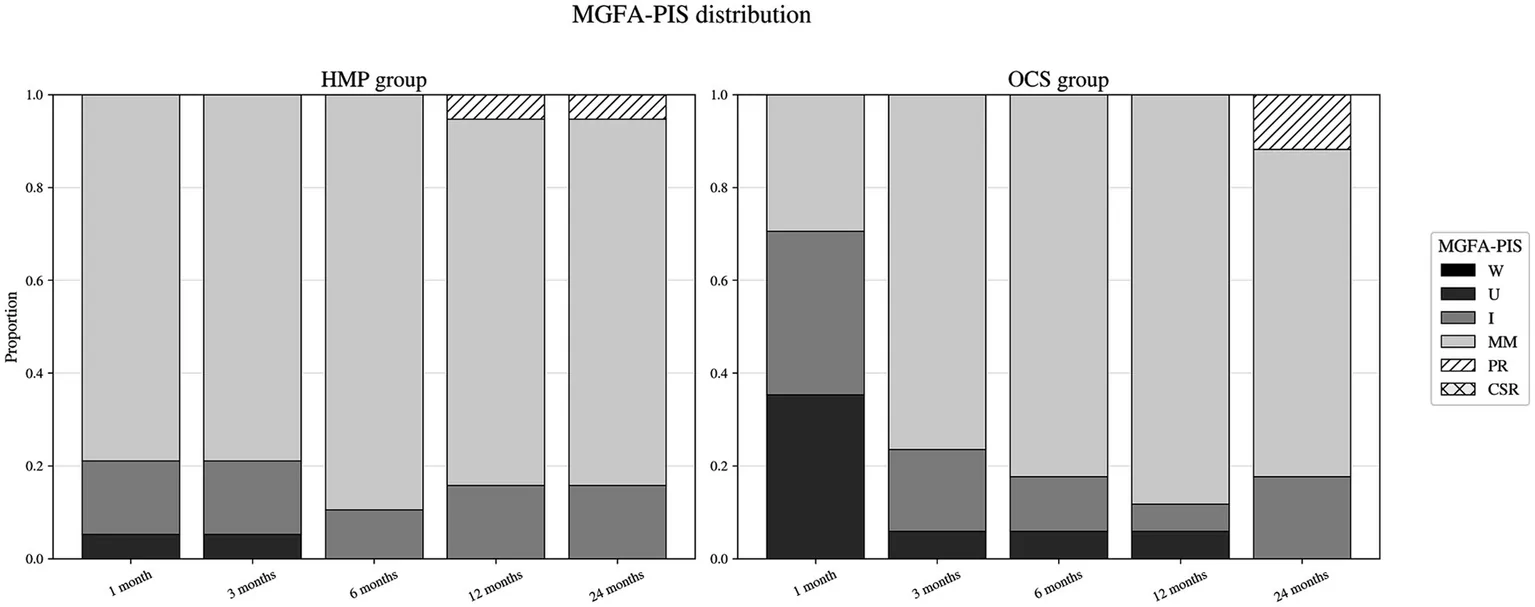
Distribution of MGFA postintervention status after immunotherapy. Stacked bar charts show the distribution of Myasthenia Gravis Foundation of America Postintervention status in the HMP and OCS groups at each follow-up time point. Bars represent the proportion of patients in each MGFA-PIS category. HMP, high-dose methylprednisolone pulse therapy; OCS, oral corticosteroid therapy; W, worse; U, unchanged; I, improved; MM, minimal manifestations; PR, pharmacological remission; CSR, complete stable remission.

The oral PSL dose did not differ significantly between the groups at any time point during follow-up. At 24 months, the mean oral PSL doses were 3.053 ± 2.094 mg/day in the HMP group and 3.235 ± 2.107 mg/day in the OCS group (*p* = 0.796). Initial worsening following HMP was observed in only two patients, and no significant between-group difference was observed in the frequency of PSL-related adverse events (Tables 3, 4).

**Table 3:** Oral prednisolone doses over 24 months following immunotherapy.

Group	Baseline	1 month	3 months	6 months	12 months	24 months
HMP group (*n* = 19)	n.p.	4.947 ± 1.471	4.789 ± 1.273	4.579 ± 1.465	3.947 ± 1.649	3.053 ± 2.094
OCS group (*n* = 17)	5.294 ± 1.213	5.294 ± 1.213	5.118 ± 0.928	4.176 ± 1.776	4.118 ± 1.691	3.235 ± 2.107
*p*-value	n.p.	0.444	0.3798	0.4668	0.7621	0.7962

Values are expressed as the mean ± standard deviation. *p*-values were calculated using the Welch’s *t*-test at each time point. HMP, high-dose methylprednisolone pulse therapy; OCS, oral corticosteroid therapy; n.p., not prescribed.

**Table 4:** Initial worsening and prednisolone-related adverse events.

Outcome	HMP group (*n* = 19)	OCS group (*n* = 17)	*p*-value
Initial worsening, *n* (%)	2 (11)	0 (0)	0.487
PSL-related adverse events, *n* (%)	4 (21)	8 (47)	0.158

Values are expressed as *n* (%). *p*-values were calculated using Fisher’s exact test. HMP, high-dose methylprednisolone pulse therapy; OCS, oral corticosteroid therapy; PSL, prednisolone.

## 4 Discussion

In the present study, longitudinal analysis of ocular MG-ADL scores demonstrated a significant group-by-time interaction, indicating different trajectories of ocular symptom improvement between the HMP and OCS groups. Although the HMP group had more severe ocular symptoms at baseline, the model-estimated ocular MG-ADL score decreased markedly by 1 month and was significantly lower than that in the OCS group after adjustment for multiple comparisons. From 3 months onward, ocular MG-ADL scores remained low in both groups, with no significant between-group differences. These findings suggest that the principal clinical benefit of early HMP may be more rapid improvement in ocular symptoms during the initial phase of treatment rather than superior long-term symptom control. The higher baseline ocular MG-ADL and diplopia scores in the HMP group may reflect confounding by indication and greater room for improvement, while the numerically shorter interval from symptom onset to immunotherapy may also have influenced early symptom reversibility. Nevertheless, the distinct longitudinal pattern observed in the mixed-effects analysis supports an association between early HMP and more rapid ocular symptom improvement.

The shorter time to achievement of MSE, a secondary outcome, in the HMP group is consistent with the reports of previous studies. These studies showed that early intravenous methylprednisolone (IVMP) accelerates the achievement of MM or MM with low-dose PSL in patients with ocular MG. Using data from the Japanese MG registry, Uzawa et al. reported that patients who received early IVMP achieved MM and MM-5 mg earlier and more frequently than those who did not (8). Similarly, Yorozu et al. demonstrated that IVMP induction increased the proportion of patients reaching MM or better as early as 1 month. This favorable treatment status was subsequently maintained for up to 12 months with low-dose immunotherapy (9). Taken together, the clinical value of HMP extends beyond early reductions in ocular MG-ADL score by facilitating earlier achievement of minimal symptom burden. Accordingly, the principal benefit of HMP may be more rapid improvement in ocular symptoms during the early phase of treatment rather than superior long-term clinical status. As ocular symptoms, particularly diplopia and ptosis, substantially impair quality of life (14–16), early symptom relief is clinically meaningful from the perspectives of functional outcomes and treatment satisfaction. Therefore, HMP may be considered for patients requiring rapid symptom control or as rescue therapy for abrupt worsening of ocular symptoms. From a practical perspective, HMP may also be readily incorporated into routine clinical practice as it is a conventional corticosteroid-based treatment that can be integrated into oral PSL-based management. In addition, HMP is relatively inexpensive in the Japanese healthcare setting (8). Thus, HMP may represent a practical induction therapy for patients requiring rapid improvement of ocular symptoms, although its applicability should be considered in the context of local healthcare resources and treatment settings.

Previous studies have suggested that HMP is particularly effective for diplopia (10, 17, 18). In the present study, the HMP group included a higher proportion of patients with more severe diplopia at treatment initiation, which may have influenced the decision to administer HMP due to the substantial impact of diplopia on quality of life. Notably, rapid improvement following HMP was observed in diplopia and ptosis (Supplementary Table S2). It has been reported that pyridostigmine tended to be more effective for ptosis than for diplopia (19) and patient-reported diplopia burden was strongly associated with MG-related quality of life (16). Therefore, it is hypothesized that the potential benefit of rapid symptom relief through HMP is particularly clinically meaningful for patients who experience persistent or disabling diplopia despite standard treatment.

No significant between-group differences were observed in the oral PSL dose at any time point; therefore, the present study did not demonstrate a steroid-sparing effect of HMP. Corticosteroids were tapered as much as possible in all patients according to our institutional policy, in line with recent Delphi-based recommendations and current international treatment concepts (20). While the present study did not examine the cumulative exposure to oral corticosteroids, it is conceivable that the observed differences between the groups were constrained due to the relatively low exposure levels of corticosteroids in both groups.

Regarding safety, treatment-related worsening is a recognized concern with corticosteroid therapy in MG, particularly in patients with generalized disease and high baseline disease activity (13). Previous studies have suggested that initial worsening following IVMP is generally transient and manageable with appropriate monitoring (21). As muscle weakness in ocular MG is confined to ocular muscles, including the extraocular muscles and levator palpebrae superioris, the risk of severe exacerbation may be lower than that in generalized MG. Consistent with this observation, Ozawa et al. reported no initial worsening or secondary generalization during a 6-month follow-up after IVMP therapy (10), and Yorozu et al. reported no serious adverse effects following IVMP induction (9). In the present study, initial worsening occurred in 2 of the 19 patients treated with HMP (11%); however, neither developed myasthenic crisis nor required intensive treatment, and both subsequently achieved favorable clinical outcomes. Despite the study’s limited sample size and inconclusive findings, the HMP appeared to be generally well tolerated under appropriate monitoring conditions. Prior to the initiation of OMG treatment, it is imperative to elucidate to patients the potential benefits, including the rapid alleviation of symptoms, as well as the risk of initial exacerbation. Furthermore, the appropriateness of the HMP must be evaluated on a case-by-case basis.

This study is not without its limitations. First, owing to the retrospective single-center design, treatment allocation was not standardized. Moreover, the possibility of selection bias could not be excluded. Second, although diplopia and ptosis subscores were available, ocular symptoms were assessed without objective quantitative measurements of ptosis or ophthalmoplegia. Vision-specific quality-of-life measures and quantitative assessments of social or occupational functioning were also not systematically available. Finally, establishing a cohort with persistent ocular MG is inherently challenging as many patients who initially present with ocular symptoms subsequently develop generalized MG, most commonly within the first 2 years after disease onset (3). To address this issue, the analysis was restricted to patients who were followed for 2 years after initiation of immunotherapy and remained classified as having ocular MG throughout the follow-up period, thereby approximating a cohort with persistent ocular MG.

## 5 Conclusion

HMP may be a useful induction option for selected patients with ocular MG when rapid improvement of functionally significant ocular symptoms is required, provided that the potential benefit is weighed against the risk of initial worsening. Its principal advantages appear to be earlier symptom relief and earlier achievement of MSE, rather than superior long-term clinical outcomes. Further prospective studies employing objective ocular muscle assessments and quality of life measures are warranted to evaluate long-term prognosis, cumulative corticosteroid exposure, and the patients most likely to benefit from HMP.

## Data Availability

The raw data supporting the conclusions of this article will be made available by the authors, without undue reservation.
