# Supplementary material for: Early high-dose intravenous methylprednisolone is associated with faster symptom improvement in persistent ocular myasthenia gravis: a single-center retrospective study
Source: Front Neurol. 2026 Oct 1;17:1956054. doi: 10.3389/fneur.2026.1956054 (PMC13670919; doi:10.3389/fneur.2026.1956054)
Supplement: Supplementary file 1 [file Table_1.DOCX]

**supplementary table**

Supplementary table S1　MSE outcomes

Supplementary table S2　Changes in diplopia and ptosis scores

Supplementary table S3　Patients with secondary generalization

Supplementary table S4　HMP administration details
